# Development of non-invasive biomarkers for pre-eclampsia through data-driven cardiovascular network models

**DOI:** 10.1038/s41598-024-72832-y

**Published:** 2024-10-04

**Authors:** Claudia Popp, Jason M. Carson, Alex B. Drysdale, Hari Arora, Edward D. Johnstone, Jenny E. Myers, Raoul van Loon

**Affiliations:** 1 Biomedical Engineering Simulation and Testing Lab, Department of Biomedical Engineering, Faculty of Science and Engineering, Swansea University, Swansea, SA1 8EN UK; 2 Division of Developmental Biology, Maternal and Fetal Health Research Centre, Faculty of Medicine Biology and Health, University of Manchester, Manchester, UK

**Keywords:** Uterine doppler waveforms, Pregnancy, Pulse wave velocity, Hypertension, Machine learning, Clinical diagnosis, Digital twin, Computational models, Statistical methods, Pre-eclampsia

## Abstract

Computational models can be at the basis of new powerful technologies for studying and classifying disorders like pre-eclampsia, where it is difficult to distinguish pre-eclamptic patients from non-pre-eclamptic based on pressure when patients have a track record of hypertension. Computational models now enable a detailed analysis of how pregnancy affects the cardiovascular system. Therefore, new non-invasive biomarkers were developed that can aid the classification of pre-eclampsia through the integration of six different measured non-invasive cardiovascular signals. Datasets of 21 pregnant women (no early onset pre-eclampsia, *n* = 12; early onset pre-eclampsia, *n* = 9) were used to create personalised cardiovascular models through computational modelling resulting in predictions of blood pressure and flow waveforms in all major and minor vessels of the utero-ovarian system. The analysis performed revealed that the new predictors *PPI* (pressure pulsatility index) and *RI* (resistance index) calculated in arcuate and radial/spiral arteries are able to differentiate between the 2 groups of women (t-test scores of *p* < .001) better than *PI* (pulsatility index) and *RI* (Doppler calculated in the uterine artery) for both supervised and unsupervised classification. In conclusion, two novel high-performing biomarkers for the classification of pre-eclampsia have been identified based on blood velocity and pressure predictions in the smaller placental vasculatures where non-invasive measurements are not feasible.

**Supplementary Information:**

The online version contains supplementary material available at 10.1038/s41598-024-72832-y.

## Introduction

Pre-eclampsia is one of the most common hypertensive disorders and is one of the leading causes of maternal mortality and morbidity^1,2^ and can be categorised into early-onset pre-eclampsia (< 34 weeks) and late-onset pre-eclampsia. Pre-eclampsia‘s pathophysiology is not fully understood but is linked to abnormal placentation in early pregnancy, leading to clinical symptoms such as elevated blood pressure and proteinuria (> 140/90 mmHg and > 0.3 g)^2–5^. Abnormal placentation involves deficient extravillous cytotrophoblast invasion of spiral arteries, hindering vascular remodelling and resulting in highly resistive vessels^2,6^. Doppler ultrasound-derived Resistance Index (RI) and Pulsatility Index (PI) measure increased resistance in uterine arteries^7–14^ correlating with later pre-eclampsia onset, however, the positive predictive values are around 50% indicating that many women with elevated indices do not develop the disease^15^.

Maternal arterial function is explored as a potential biomarker due to the recognition of arterial stiffness as a risk factor in assessing cardiovascular diseases^16^. Investigations involve monitoring pulse wave velocity (PWV), larger artery diameter, pulse pressure and more^17,18^. Hypertensive women can exhibit increased PWV^19–22^ and associations with altered aortic wall elastic properties resulting in increased diameter and decreased vascular compliance were found^23^.

Although all these metrics show evidence of a relation between arterial stiffness and pre-eclampsia, they are not enough to capture the spatial variations throughout the maternal cardiovascular system. As a result, computational modelling approaches have been developed to provide a more in-depth understanding of these variations during pregnancy, especially for the utero-ovarian system and its properties such as the resistances and compliances. The benefit of these models is that they can predict values of pressure and flow downstream of the point measured due to the wave propagation along each vessel via 1D modelling^24^. This will help understand the cardiovascular responses in the vessels downstream of the uterine artery, locations that are challenging to measure^25^.

This study explores alternative biomarkers from maternal circulation mechanics utilising the model developed by Carson et al.^26^. The new biomarkers will be assessed using a clinical cohort of high risk women for early onset pregnancy disease (no early onset pre-eclampsia = 12, early onset pre-eclampsia = 9). The use of personalised computational models is advantageous as they can predict patient-specific solutions.

##  Materials and methods

All methods were performed in accordance with the relevant guidelines and regulations as stipulated by University of Manchester. This was a retrospective pilot study based on data from St. Mary’s Hospital, Manchester, UK, collected with the approval of NHS Research Ethics Committees (RECs). Informed consent was received from all participants in this study.

### A computational model of the maternal circulation and wave reflection

**Fig. 1:**
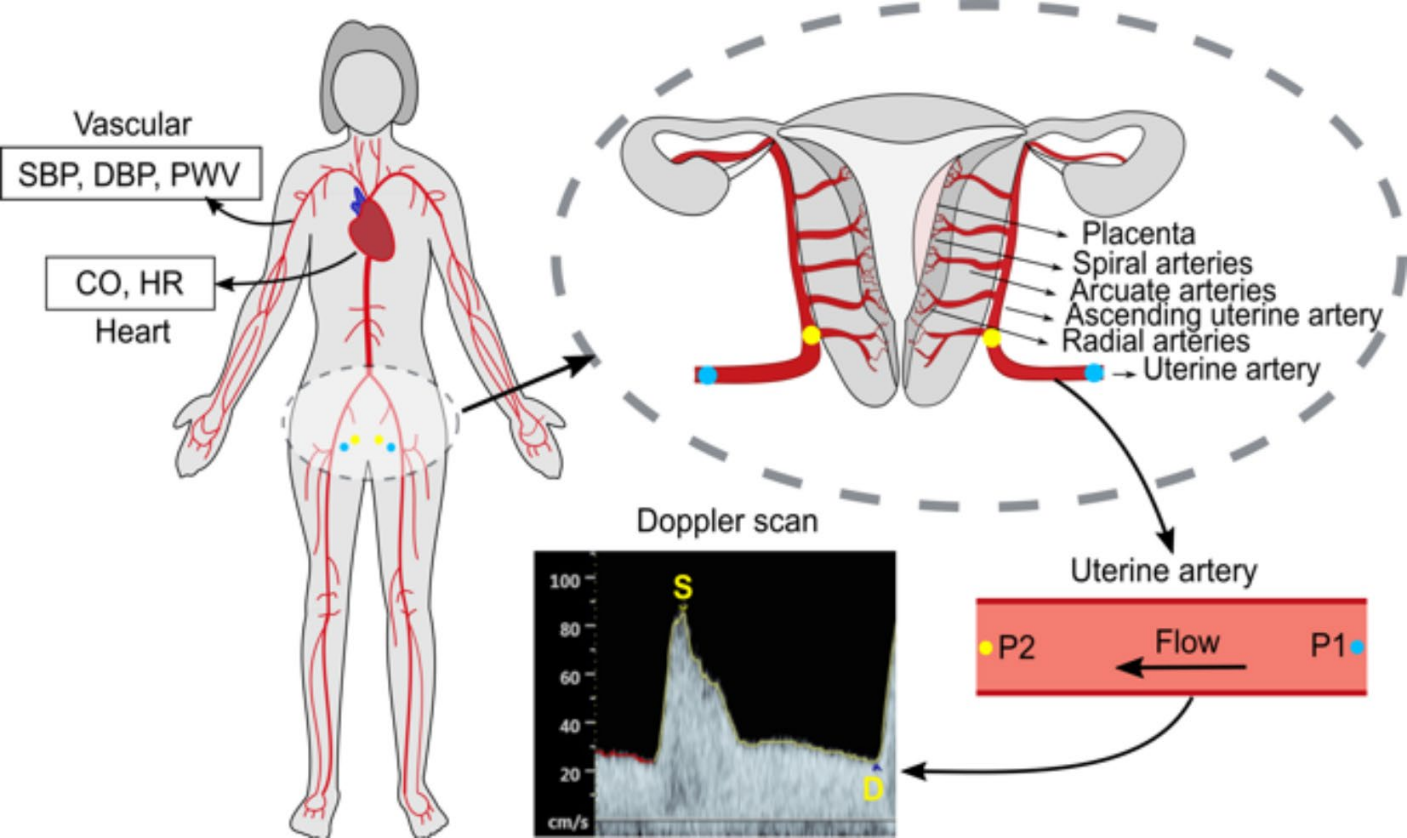
Maternal arterial network model (left). The pressure upstream of the uterine artery (P1, blue dots) are calculated using global maternal measurements of heart and vasculature together with the uterine Doppler waveform (S – peak systolic velocity, D – end-diastolic velocity) resulting in the prediction of downstream pressure (P2, yellow dots); P1 – pressure at start of uterine artery, P2 – pressure at end of uterine artery.

This study utilizes models by Carson et al.^24,26,27^ comprising larger arteries, veins, vascular beds (including organs and capillary systems), and a heart model. Larger vasculatures are modelled for wave propagation using one-dimensional flow models, while electric circuit models describe the vascular beds. Figure 1 demonstrates how the maternal arterial system, utero-ovarian vasculature, and non-invasive measurements are employed to create personalised models.

Parameters as SBP, DBP, PWV, CO, HR and blood flow in the uterine artery (measured using standard methods such as blood pressure monitors, Doppler scan etc.) are used to converge the computational model (Fig. 1).

The resulting personalised models form a “digital twin” of each patient that characterises the patient’s cardiovascular physiology. It can provide predictions of pressures/flow waveforms throughout the larger vasculature, but also in the arcuate/spiral arteries in the utero-ovarian vasculature (Fig. 1).

### Patient characteristics

This was a case control pilot study amongst a high risk cohort (see Table 1). To determine an appropriate sample size, a one-tailed t-test with a typical power of 80% was employed. Given the integration of various measurements into the model, the effect size was anticipated to be large (equal to 1), whilst alpha was chosen to be 0.1. From this a target sample size of 10 for each of the groups was estimated, which led to the final study groups of 9 and 12 for whom the required combination of measurements were taken.

Women were selected from a cohort attending high-risk clinics, where they were referred due to risk factors for developing pre-eclampsia and fetal growth restriction (FGR)^28^. Participants were randomly chosen from the clinic database, ensuring a complete dataset of measurements (required for running the computational model, see Table 1) was available. The two groups outcomes were defined as following: NPE group contains women with no medical conditions or late onset pre-eclampsia or late onset FGR while PE contains women that were diagnosed with early onset pre-eclampsia or early onset FGR. Diagnosis of pre-eclampsia was made in accordance with ISSHP guidelines^29^.

**Table 1:** The measurements were obtained during routine visits. SD – standard deviation; FDIU – fetal death in utero, NPE – no early onset pre-eclampsia group, PE – early onset pre-eclampsia group* (*= with 2 early onset FGR), GA – gestational age.

	NPE (*n* = 12) Mean ± SD	PE (*n* = 9) Mean ± SD
*Pregnancy measurements*		
GA at measurement (weeks)	23.3 ± 0.7	25.2 ± 2
Parity	1.7 ± 1.4	0.7 ± 0.9
BMI	29.6 ± 7.3	28.3 ± 3.2
SBP (mmHg)	133.9 ± 13	140.9 ± 17.6
DBP (mmHg)	89.6 ± 8.2	90.7 ± 12.2
HR (beats/min)	91.6 ± 11.9	81.9± 11.3
CO (L/min)	4.6 ± 1.4	6.1 ± 1.1
PWV (m/s)	7.3 ± 1.7	8.9 ± 1.5
Age (years)	32.2 ± 3.7	34.1 ± 4.6
Weight (kg)	79 ± 22.6	75.8 ± 8.5
PI	0.9 ± 0.2	1.7 ± 0.7
RI	0.5 ± 0.1	0.7 ± 0.1
*Pregnancy outcome*		
GA at birth (weeks)	38.3 ± 1.7	27.9 ± 2.4
Birth Weight (g)	2933.7 ± 683.4	540.6 ± 220.5
Complications (by birth)	2 Late FGR, 3 Late onset PE	7 Early onset PE, 2 Early FGR
Adverse pregnancy outcome (%)	0%	11% neonatal death, 33% FDIU
*Medical history*		
History of hypertension	83.3% Yes	22.2% Yes
*Demographics*		
Ethnic group	2 African (Black or Black British), 1 Pakistani (Asian or Asian British), 1 Caribbean (Black or Black British), 5 British (White), 2 any other ethnic group	2 African (Black or Black British), 2 Pakistani (Asian or Asian British), 4 British (White), 1 any other ethnic group

### Defining potential classifiers

The new biomarkers proposed for the assessment of pre-eclampsia were derived using Buckingham Pi theorem and can be found in Eqs. 1–6 (${\pi _1}$, ${\pi _2}$, ${\pi _3}$, ${\pi _4}$, ${\pi _5}$ and ${\pi _6}$). The parameters that were used to compose the dimensionless terms are uterine vessel resistance ${R_{ut}}$, Stroke volume *SV*, Cardiac output *CO*, Systemic compliance ${C_{syst}}$, Peripheral resistance ${R_{periph}}$, Pulse Wave Velocity *PWV*, Systolic Blood Pressure ${P_{syst}}$, Pulse pressure $\Delta {P_{pulse}}$, Aortic area *A*.

1 $${\pi _1}=\frac{{{R_{ut}}}}{{{R_{periph}}}}$$

2 $${\pi _2}=\frac{{S{V^2}}}{{{A^3}}}$$

3 $${\pi _3}=\frac{{CO\,{R_{periph}}}}{{{P_{syst}}}}$$

4 $${\pi _4}=\frac{{{C_{syst}}\,{P_{syst}}}}{{{A^{\frac{3}{2}}}}}$$

5 $${\pi _5}=\frac{{{R_{periph}}\,A\,PWV}}{{{P_{syst}}}}$$

6 $${\pi _6}=\frac{{\Delta {P_{pulse}}}}{{{P_{syst}}}}$$

Two further dimensionless terms are proposed based on the pressure and velocity in the utero-ovarian vessels such as ascending uterine artery, arcuate artery and radial/spiral arteries (the radial and spiral arteries were modelled together so when referring to radial arteries or subscript *rad*, spiral arteries were also included). The formulation of pressure index for the utero-umbilical system was introduced by Adamson et al.^30^. An alternative metric proposed clinically is the pulmonary artery pulsatility index (PAPi) which was defined as the pulmonary artery pulse pressure (PAP) divided by the right atrial pressure (RAP)^31^. Here we propose pressure pulsatility index, *PPI*, as:

7 $$PPI=\frac{{{P_{max}} - {P_{min}}}}{{{P_{mean}}}}$$

where ${P_{min}}$ is the minimum pressure in the selected utero-ovarian vessel and ${P_{max}}$ is the maximum pressure during the cardiac cycle. *PPI* relates pulsatility to pressure and should not be confused with the *PI* which captures the pulsatility of the velocity.

*RI* has the generic form shown in Eq. 8. The *RI* calculated from the Doppler scan will be noted as *RI* and the *RI* calculated from the computational model will be noted with the appropriate subscript.

8 $$RI=\frac{{{V_{max}} - {V_{min}}}}{{{V_{max}}}}$$

### Classification analysis

The terms stated above were used in a binary classification problem (PE and NPE). The analysis was performed using the Classification Learner App in MATLAB R2021b. To analyse the data both Logistic Regression (supervised learning) and k-means clustering (unsupervised learning) were used. The supervised learning training sample size was 19 and testing sample size was 2, where a two-fold cross-validation was used during training. This process was repeated 5 times to reduce bias. The purpose of the supervised classification is to assess the features’ classification performance while the unsupervised machine learning focuses on the features’ ability to classify the two groups in a bias-free manner.

The dimensionless terms, *PPI*, *RI*, ${\pi _1}$, ${\pi _2}$, ${\pi _3}$, ${\pi _4}$, ${\pi _5}$, ${\pi _6}$, ${\pi _7}$ and the clinical parameters *PI*, *RI*, *DBP* and *SBP* were selected as classifiers. The metrics used to assess the classification were: A – accuracy (%), 95% CI – confidence interval (%), SE – sensitivity (%), and SP – specificity (%). Accuracy is defined as the number of correct predictions divided by the total number of predictions.

##  Results

The results presented only display the right side of the circulation (*RI*, *PI*, and *PPI*) as the difference between right and left side was not statistically significant.

Table 2 displays model accuracies for individual features, revealing that computational indices *PPI* and *RI* outperform *PI* and *RI* from Doppler scans. T-test scores confirm that *SBP/DBP* are inappropriate diagnostic criteria for early-onset pregnancy diseases in high-risk women. Effect size analysis (Cohen’s d) supports this, with low scores for *SBP* and *DBP* and high scores for *PI*, *PPI* and *RI* indicating their diagnostic significance.

**Table 2:** Classification analysis results of individual features where supervised ML was performed using logistic regression from the Classifier App while unsupervised ML was performed using k-means; the results for the supervised ML show the mean of all algorithms that were trained/tested. A – accuracy (%), 95% CI – confidence interval (%) SE – sensitivity (%), SP – specificity (%), AUC – area under the ROC curve.

Features	t-test (*p*-score)	Supervised ML					Unsupervised ML			
		Training + Testing								
		AUC (Training only)	A	CI	SE	SP	A	CI	SE	SP
${\pi _1}$	0.001	0.85	85.7	15.0	84.6	87.5	76.2	18.2	88.9	66.7
${\pi _2}$	0.0099	0.86	67.7	20.2	72.7	60.0	76.2	18.2	100	64.3
${\pi _3}$	0.3146	0.61	33.3	20.2	42.8	0.0	57.1	21.2	71.4	50
${\pi _4}$	0.0055	0.93	85.7	15.0	90.9	80.0	76.2	18.2	100	64.3
${\pi _5}$	0.0244	0.86	66.7	20.2	66.7	66.7	66.7	21.2	85.7	57.1
${\pi _6}$	0.3489	0.24	52.4	21.4	55.6	33.3	47.6	21.4	60	45.4
$PP{I_{arc}}$	0.0006	0.86	81.0	16.8	78.6	85.7	85	15	84.6	87.5
$R{I_{arc}}$	10^−6^	0.89	90.5	12.5	91.7	88.9	95.2	9.1	92.3	100
$PP{I_{rad}}$	0.0104	0.8	81.0	16.8	78.6	86.7	80.9	16.8	78.6	85.7
$R{I_{rad}}$	0.0006	0.84	85.7	15.0	84.6	87.5	90.5	12.5	91.7	88.9
$PI$	0.0006	0.89	81.0	16.8	83.3	77.8	85.7	15	80	100
$RI$	0.0011	0.85	76.2	18.2	81.8	70.0	80.9	16.8	90	72.7
*SBP*	0.3327	0.4	47.6	21.4	52.9	75.0	52.4	21.4	50	55.5
*DBP*	0.8195	0.57	52.4	21.4	55.0	0.0	57.1	21.2	33.3	75

**Fig. 2:**
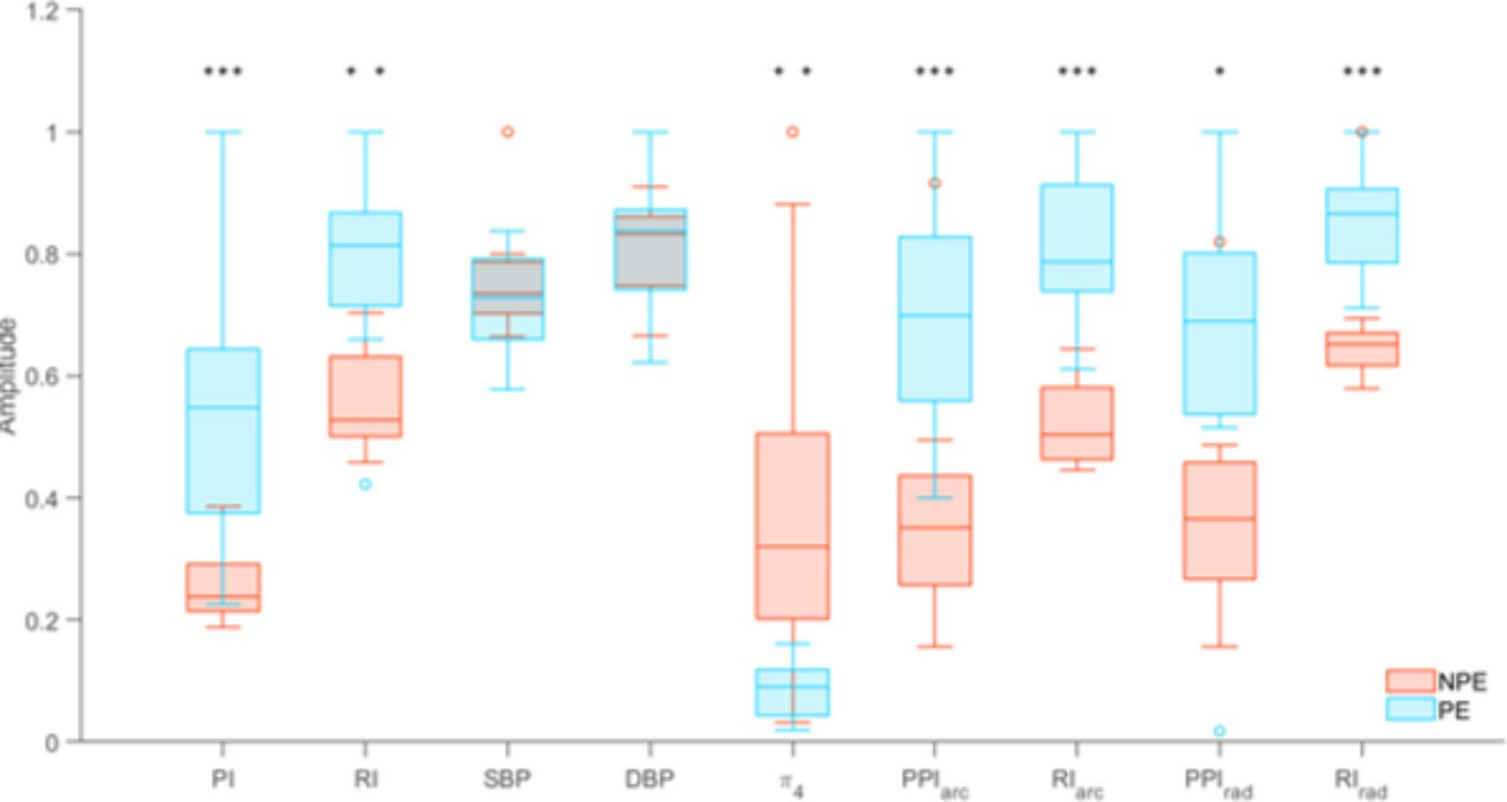
Comparison of NPE and PE selected features using boxplots were the median, minimum and maximum ranges are displayed. Main outliers: PE8 for RI; NPE7 for SBP; NPE9 for $PP{I_{arc}}$, $PP{I_{rad}}$, $R{I_{rad}}$; *p-score < 0.05, **p-score < 0.01, ***p-score < 0.001.

The values presented in Fig. 2 were normalised within the range [0,1] and the p-scores highlighted. Figure 2 depicts boxplot comparisons between the two groups for the best performing features. For the PE group, the medians of *PI*, *RI*, *PPI*, and $RI$ were significantly higher than the NPE group, whilst *SBP* and *DBP* showed very similar medians between the NPE and PE groups.

The next features that resulted in high accuracy are ${\pi _1}$ and ${\pi _4}$ which showed accuracies of over 85% (higher than *PI* and *RI*) but the accuracy dropped considerably for unsupervised classification. Even so, this result for ${\pi _4}$ suggests that the aortic area could be related to changes in the uterine region, as found by Orabona et al.^23^ and the calculated resistance of the uterine arteries of ${\pi _1}$ had a significant impact on the classification of the groups. The features were also paired with each other to search for stronger biomarkers, but the classification results did not improve compared to those presented in Table 2. For the unsupervised classification, $R{I_{arc}}$ showed an accuracy of 95.2%. Besides $R{I_{arc}}$, all other biomarkers including *PI* and *RI* classified two or more patients incorrectly.

**Fig. 3:**
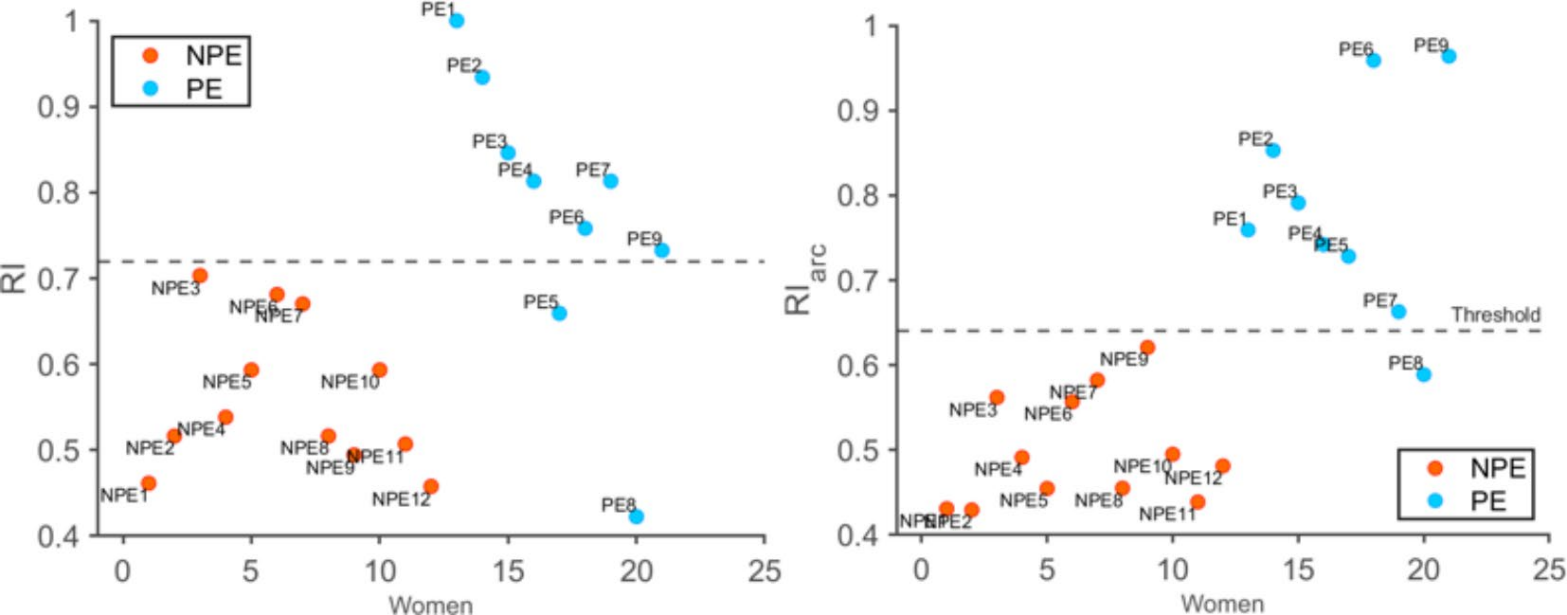
RI values for the two groups with threshold of 0.72 (left), $R{I_{arc}}$ values for the two groups with threshold of 0.64 (right).

Lastly, a threshold value was calculated for $R{I_{arc}}$ and compared to the same biomarker calculated in the uterine artery (*RI*) for a direct comparison. The plots clearly show that the two groups are more clustered together for $R{I_{arc}}$ compared to *RI* (Fig. 3).

## Discussion

The data-driven modelling approach taken in this work demonstrated that integrating key physiological measurements into computational models allows us to define a range of new metrics for classifying placental disease-complicated pregnancies. The newly defined metrics *PPI* and $RI$ in the arcuate and spiral arteries seem more accurate classifiers of “at risk” pregnancies (e.g. early-onset pre-eclampsia) than standard measurements such as *SBP/DBP*. The modelling approach has allowed the integration of multivariate data to develop personalised models and to use them to predict pressures/flows in locations where measurement is not possible (i.e. arcuate or radial/spiral arteries).

It is worth noting that NPE9 was the most misclassified woman as a false positive. NPE9 shows a *PWV* of 12.3 m/s, *HR* of 117 beats/min and pressure measurements of 146/95 mmHg which is considerably higher than the mean of the NPE group. When calculating the π terms, it was found that this specific woman had the highest BMI (= 44.1) in the group with a model-predicted aortic cross-sectional area value that sits in the PE group range. Additionally, the medical history indicates a diagnosis of chronic hypertension and blood pressure measurements of 138/84 mmHg pre-pregnancy. This could explain why the new biomarkers fail to classify this specific woman in the NPE group. When looking at other classifiers that have a lower relation to pressure like ${\pi _1}$, the result for this woman was similar to the group range. It would be interesting to understand the relationship between high BMI and pre-eclampsia in more depth as obesity is a known risk factor for pre-eclampsia^32–35^ but in this case, the high BMI did not lead to early onset pre-eclampsia.

The most common false negative was PE8. *RI*, $R{I_{arc}}$, and $PP{I_{rad}}$ identified this woman as a false negative. Looking at it in more detail, the measurements taken seem to be ordinary with only a low *DBP* (69 mmHg compared to the median of the group of 93 mmHg) and small CO (4.8 L/min). Regarding the Doppler scans, the end-Diastolic Velocity for both left and right were significantly higher than the rest of the group which resulted in a lower *RI*. Interestingly, it was observed that ${\pi _4}$ did not misclassify PE8 as it uses the global maternal parameters rather than the local uterine artery velocity. The medical history did not reveal any hypertension history. One possibility for this outcome could be that the woman might have a different phenotype of placental disease.

There is a general consensus that the Doppler indices *RI* and *PI* are related to the vascular resistance in the uterine circulation and pre-eclamptic women will show an increase in *RI/PI* due to the vascular remodelling from high resistance to lower resistance vessels^36^. This was observed in Tables 1 and 2 where the classification results display a great accuracy for *PI* with AUC of 0.89. The PPI and RI biomarkers which are derived from the downstream uterine circulation show great results with similar AUC values (from 0.8 to 0.89). This suggests that the changes seen in early onset pre-eclampsia are related to the resistance in uterine circulation due to vascular remodelling and is also highlighted in ${\pi _1}$ classification results (p-score of 0.001 & classification accuracy of 85%) which is a biomarker consisting of the computational model’s calculated uterine resistance, ${R_{ut}}$, and peripheral resistance, ${R_{periph}}$.

This illustrates that the newly found *PPI* and *RI* calculated in the smaller vasculatures show great potential when assessing women with a high risk of developing early onset pre-eclampsia, however, they require more testing in bigger populations to confirm their ability. Their current lack of statistical power in this pilot study is illustrated by the DeLong p-values found in the Supplementary Material s7^37^. Sedaghati et al.^38^ highlighted the advantages of employing an intricate mathematical model customised for pregnancy in analysing the impact of pre-eclampsia, alongside underscoring the significance of *PI* in evaluating early or late onset pre-eclampsia. Exploring the effect of *PPI* and *RI* computed within the smaller vasculature on a sophisticated mathematical model capable of replicating maternal physiology more authentically would offer further valuable insights.

Biomarker ${\pi _4}$ demonstrates an accuracy of 85.7% and AUC of 0.93 but faced challenges due to low specificity, resulting in more false positives (classifying NPE women as PE women). This is visualised in Fig. 2 where the NPE group’s lower quartile nearly overlaps the PE group’s range. The inclusion of aortic area, *A*, *SBP* and ${C_{syst}}$ in ${\pi _4}$ suggests a difference in aortic area for early onset pre-eclamptic women, consistent with findings by Spaanderman et al.^39^ that presented hypertensive women with a history of pre-eclampsia had lower compliance and increased aortic areas. In a study performed on gestational age > 33 weeks, it was found that PWV was higher in pre-eclamptic women compared to hypertensive ones^40^. Conversely, biomarkers involving PWV or SV did not show higher classification accuracy than ${\pi _4}$, indicating PWV’s inadequacy for early classification of early onset pre-eclampsia (< 28 weeks).

Based on this pilot study, the predictive ability of our biomarkers seems promising, but it’s too early to to draw a strong conclusion about the possibility of *PPI* or *RI* predicting early onset pre-eclampsia or how they perform in late pregnancy. These limitations can be explored in future work.

Finally, it should be noted that the modelling framework used is currently computationally intensive, and opportunities for simplification are currently explored. Future directions could be around the differential diagnosis of disorders such as FGR, pregnancy induced hypertension, and placental dysfunction by adding weights to certain parameters that are heavily affected by a given disease. Lastly, the model could be used to further analyse flow and pressure waveform shapes and how their form is related to pregnancy complications.

##  Conclusion

The modelling framework proposed gives an insight in how various clinical measurements are related, which makes it a useful tool to study the differences between these patient groups. *PPI* and *RI* calculated in the smaller uterine vasculature have shown better accuracy in the classification of women with a high risk of developing early onset pre-eclampsia as part of the retrospective study. The results were comparable to *PI* and *RI* calculated in the Doppler scan meaning usage of the computational model provides insightful information of the cardiovascular mechanics of the pregnant woman. Clinical methods are not able to properly assess the blood flow in the smaller vasculature which makes the usage of computational model and deriving new biomarkers from it a novelty.

## Supplementary material

Below is the link to the electronic supplementary material.

Supplementary Material 1.

## Data Availability

The full data can be found in the Supplementary Material.
