# Supplementary material for: Development of non-invasive biomarkers for pre-eclampsia through data-driven cardiovascular network models
Source: Sci Rep. 2024 Oct 4;14:23144. doi: 10.1038/s41598-024-72832-y (PMC11452701; doi:10.1038/s41598-024-72832-y)

**Supplementary Material**

**Development of non-invasive biomarkers for pre-eclampsia through data-driven cardiovascular network models**

**Authors:** Claudia Popp, Jason M. Carson, Alex B. Drysdale, Hari Arora, Ed Johnstone, Jenny Myers, Raoul Van Loon

**Supplementary Table s1: Full patient measurement data**

| **Participant** | **Height** | **Weight** | **Week** | **SBP** | **DBP** | **HR** | **CO** | **PWV** | **Birth Weight** | **BMI** | **Lt Ut-PI** | **Rt Ut-PI** | **Lt Ut-RI** | **Rt Ut-RI** |
| --- | --- | --- | --- | --- | --- | --- | --- | --- | --- | --- | --- | --- | --- | --- |
| NPE1 | 172 | 95 | 169 | 146 | 96 | 91 | 6.6 | 6.3 | 2117 | 32.1 | 1.11 | 0.6 | 0.62 | 0.42 |
| NPE2 | 155 | 50 | 170 | 107 | 74 | 94 | 3.5 | 5.8 | 2052 | 20.8 | 0.79 | 0.68 | 0.52 | 0.47 |
| NPE3 | 169 | 114 | 156 | 145 | 93 | 88 | 4.9 | 7 | 3260 | 39.9 | 0.62 | 1.14 | 0.44 | 0.64 |
| NPE4 | 166 | 79 | 168 | 122 | 87 | 76 | 5.7 | 6.7 | 3370 | 28.7 | 0.88 | 0.72 | 0.55 | 0.49 |
| NPE5 | 155 | 54 | 157 | 123 | 79 | 74 | 2.5 | 6 | 2420 | 22.5 | 0.83 | 0.86 | 0.53 | 0.54 |
| NPE6 | 158 | 76 | 167 | 133 | 95 | 96 | 4.4 | 7.5 | 1912 | 30.4 | 0.72 | 1.23 | 0.48 | 0.62 |
| NPE7 | 166 | 79 | 162 | 141 | 97 | 83 | 3.3 | 8.1 | 3240 | 28.7 | 0.57 | 1 | 0.41 | 0.61 |
| NPE8 | 164 | 44.2 | 165 | 129 | 88 | 84 | 4.2 | 7.5 | 2300 | 16.4 | 1.52 | 0.69 | 0.72 | 0.47 |
| NPE9 | 167 | 123 | 163 | 146 | 95 | 117 | 5.6 | 12.3 | 3800 | 44.1 | 1.2 | 0.63 | 0.62 | 0.45 |
| NPE10 | 160 | 74.1 | 162 | 155 | 101 | 99 | 3.7 | 5.9 | 3369 | 28.9 | 1.13 | 0.83 | 0.61 | 0.54 |
| NPE11 | 156 | 80 | 154 | 124 | 78 | 89 | 3.5 | 7.3 | 3670 | 32.9 | 0.8 | 0.8 | 0.51 | 0.46 |
| NPE12 | 165 | 80 | 167 | 136 | 92 | 108 | 7.3 | 6.6 | 3694 | 29.4 | 0.69 | 0.69 | 0.33 | 0.42 |
| PE1 | 156 | 76 | 196 | 185 | 111 | 81 | 4.7 | 12.5 | 931 | 31.2 | 2.55 | 3.19 | 0.87 | 0.91 |
| PE2 | 172 | 79 | 165 | 148 | 94 | 69 | 6.1 | 8.1 | 340 | 26.7 | 1.86 | 2.49 | 0.78 | 0.85 |
| PE3 | 153 | 77 | 154 | 127 | 77 | 79 | 7.1 | 7 | 350 | 32.9 | 2.52 | 1.91 | 0.83 | 0.77 |
| PE4 | 157 | 57 | 196 | 122 | 84 | 79 | 4.5 | 7.6 | 494 | 23.1 | 1.64 | 1.75 | 0.72 | 0.74 |
| PE5 | 165 | 65 | 159 | 148 | 90 | 81 | 7.1 | 9.3 | 260 | 23.9 | 0.84 | 1.06 | 0.60 | 0.6 |
| PE6 | 166 | 80 | 179 | 138 | 105 | 99 | 7.3 | 9.4 | 550 | 29.0 | 1.82 | 1.24 | 0.77 | 0.69 |
| PE7 | 176 | 83 | 186 | 135 | 93 | 71 | 5.7 | 7.9 | 895 | 26.8 | 1.84 | 1.54 | 0.76 | 0.74 |
| PE8 | 164 | 85 | 176 | 134 | 69 | 74 | 4.8 | 9.1 | 495 | 31.6 | 0.72 | 0.72 | 0.38 | 0.38 |
| PE9 | 166 | 80.2 | 177 | 131 | 93 | 104 | 7.3 | 9.4 | 550 | 29.1 | 1.77 | 1.77 | 0.67 | 0.67 |

**Supplementary Table s2: Participant** medical history

| **Participant** | **Age** | **Ethnic group** | **Height (Pre)** | **Weight (Pre)** | **SBP (Pre)** | **DP (Pre)** | **Complication** | **Medical history** |
| --- | --- | --- | --- | --- | --- | --- | --- | --- |
| NPE1 | 32 | African (Black or Black British) | 172 | 95 | 130 | 90 | Late FGR | Chronic hypertension |
| NPE2 | 33 | Pakistani (Asian or Asian British) | 155 | 50 | 130 | 95 | Late FGR | Renal hypertension |
| NPE3 | 36 | Caribbean (Black or Black British) | 169 | 114 | 132 | 78 | No complication | Chronic hypertension |
| NPE4 | 35 | British (white) | 166 | 79 | 122 | 80 | No complication | Chronic hypertension |
| NPE5 | 35 | British (white) | 155 | 54 | 120 | 72 | Late onset PE (34) | Chronic hypertension |
| NPE6 | 28 | African (Black or Black British) | 158 | 76 | 122 | 86 | Late onset PE (34) | Chronic hypertension |
| NPE7 | 31 | Any other ethnic group | 166 | 79 | 166 | 102 | No complication | Chronic hypertension |
| NPE8 | 36 | Any other ethnic group | 164 | 44 | 131 | 56 | Late onset PE (34) |  |
| NPE9 | 25 | British (white) | 167 | 123 | 138 | 84 | No complication | Chronic hypertension |
| NPE10 | 31 | Chinese | 160 | 74 | 150 | 80 | No complication |  |
| NPE11 | 37 | British (white) | 156 | 80 | 130 | 88 | No complication | Chronic hypertension |
| NPE12 | 27 | British (white) | 164 | 80 | 130 | 88 | No complication | Chronic hypertension |
| PE1 | 31 | African (Black or Black British) | 156 | 76 | 142 | 70 | Early onset PE | Chronic hypertension |
| PE2 | 40 | British (white) | 171 | 72 | 125 | 78 | Early onset PE |  |
| PE3 | 34 | African (Black or Black British) | 158 | 77 |  |  | Early onset PE |  |
| PE4 | 27 | Pakistani (Asian or Asian British) | 157 | 57 | 100 | 60 | Early onset PE |  |
| PE5 | 27 | British (white) | 165 | 65 | 105 | 70 | Early onset PE |  |
| PE6 | 38 | Pakistani (Asian or Asian British) | 166 | 80 | 110 | 60 | Early onset PE |  |
| PE7 | 34 | British (white) | 176 | 83 | 122 | 83 | Early onset PE | Renal hypertension |
| PE8 | 38 | British (white) | 164 | 85 | 106 | 58 | Early FGR |  |
| PE9 | 38 | Any other ethnic group | 166 | 80 | 110 | 60 | Early FGR |  |

**Supplementary Table s3: Full Values for calculating biomarkers except the ones already provided in Table 1 (normalised, results were rounded to 3 decimal places)**

| **Group** | **A** | **PWV** | **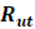** | **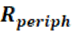** | **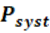** | **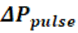** | **Compliance** | **CO** | **SV** |
| --- | --- | --- | --- | --- | --- | --- | --- | --- | --- |
| NPE1 | 0.325 | 0.509 | 0.891 | 0.339 | 0.784 | 0.676 | 0.801 | 0.904 | 0.807 |
| NPE2 | 0.227 | 0.459 | 0.176 | 0.517 | 0.576 | 0.452 | 1 | 0.479 | 0.414 |
| NPE3 | 0.296 | 0.565 | 0.566 | 0.533 | 0.781 | 0.707 | 0.63 | 0.671 | 0.62 |
| NPE4 | 0.483 | 0.532 | 0.268 | 0.465 | 0.659 | 0.473 | 0.741 | 0.781 | 0.835 |
| NPE5 | 0.171 | 0.487 | 0.105 | 1 | 0.667 | 0.614 | 0.794 | 0.342 | 0.376 |
| NPE6 | 0.353 | 0.606 | 0.384 | 0.679 | 0.723 | 0.512 | 0.576 | 0.603 | 0.51 |
| NPE7 | 0.302 | 0.655 | 0.723 | 0.919 | 0.764 | 0.593 | 0.457 | 0.452 | 0.442 |
| NPE8 | 0.352 | 0.601 | 0.266 | 0.635 | 0.694 | 0.554 | 0.577 | 0.575 | 0.556 |
| NPE9 | 0.574 | 0.918 | 0.56 | 0.53 | 0.788 | 0.689 | 0.222 | 0.767 | 0.533 |
| NPE10 | 0.184 | 0.492 | 0.564 | 0.61 | 0.851 | 0.767 | 0.745 | 0.507 | 0.416 |
| NPE11 | 0.246 | 0.585 | 0.089 | 0.745 | 0.663 | 0.63 | 0.598 | 0.479 | 0.438 |
| NPE12 | 0.362 | 0.54 | 0.419 | 0.304 | 0.741 | 0.594 | 0.769 | 1 | 0.752 |
| PE1 | 0.504 | 1 | 0.519 | 0.799 | 1 | 1 | 0.207 | 0.644 | 0.646 |
| PE2 | 0.522 | 0.645 | 1 | 0.461 | 0.807 | 0.727 | 0.487 | 0.836 | 0.984 |
| PE3 | 0.511 | 0.574 | 0.286 | 0.332 | 0.693 | 0.675 | 0.681 | 0.973 | 1 |
| PE4 | 0.45 | 0.619 | 0.494 | 0.55 | 0.657 | 0.513 | 0.569 | 0.616 | 0.634 |
| PE5 | 0.59 | 0.705 | 0.647 | 0.4 | 0.791 | 0.782 | 0.396 | 0.973 | 0.975 |
| PE6 | 1 | 0.751 | 0.511 | 0.404 | 0.754 | 0.445 | 0.334 | 1 | 0.82 |
| PE7 | 0.535 | 0.615 | 0.345 | 0.495 | 0.72 | 0.567 | 0.526 | 0.781 | 0.893 |
| PE8 | 0.38 | 0.721 | 0.378 | 0.589 | 0.713 | 0.886 | 0.414 | 0.658 | 0.722 |
| PE9 | 0.76 | 0.709 | 0.323 | 0.375 | 0.705 | 0.514 | 0.364 | 1 | 0.781 |

**Supplementary Table s4:** **Full Biomarkers values (normalised, results were rounded to 3 decimal places)**

| **Participant** | *PI* | *RI* | *SBP* | *DBP* | 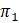 | 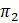 | 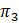 | 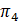 | 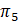 | 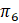 | 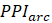 | 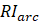 | 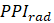 | 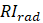 |
| --- | --- | --- | --- | --- | --- | --- | --- | --- | --- | --- | --- | --- | --- | --- |
| NPE1 | 0.188 | 0.462 | 0.784 | 0.865 | 0.069 | 0.862 | 0.720 | 0.449 | 0.129 | 0.696 | 0.494 | 0.447 | 0.446 | 0.666 |
| NPE2 | 0.213 | 0.516 | 0.665 | 0.667 | 0.161 | 0.640 | 0.779 | 0.561 | 0.189 | 0.625 | 0.199 | 0.446 | 0.229 | 0.580 |
| NPE3 | 0.357 | 0.703 | 0.719 | 0.838 | 0.354 | 0.678 | 0.782 | 0.357 | 0.206 | 0.723 | 0.317 | 0.583 | 0.324 | 0.652 |
| NPE4 | 0.226 | 0.538 | 0.800 | 0.784 | 0.198 | 0.408 | 0.998 | 0.158 | 0.372 | 0.581 | 0.439 | 0.510 | 0.470 | 0.655 |
| NPE5 | 0.270 | 0.593 | 0.746 | 0.712 | 0.184 | 0.951 | 0.840 | 0.881 | 0.210 | 0.729 | 0.190 | 0.472 | 0.199 | 0.607 |
| NPE6 | 0.386 | 0.681 | 0.670 | 0.856 | 0.345 | 0.382 | 0.977 | 0.204 | 0.394 | 0.577 | 0.319 | 0.578 | 0.305 | 0.628 |
| NPE7 | 0.313 | 0.670 | 1.000 | 0.874 | 0.369 | 0.407 | 0.913 | 0.219 | 0.462 | 0.625 | 0.377 | 0.604 | 0.335 | 0.652 |
| NPE8 | 0.216 | 0.516 | 0.762 | 0.793 | 0.113 | 0.421 | 1.000 | 0.199 | 0.417 | 0.645 | 0.393 | 0.472 | 0.396 | 0.674 |
| NPE9 | 0.197 | 0.495 | 0.697 | 0.856 | 0.301 | 0.156 | 0.965 | 0.033 | 0.887 | 0.698 | 0.916 | 0.644 | 0.820 | 1.000 |
| NPE10 | 0.260 | 0.593 | 0.789 | 0.910 | 0.503 | 1.000 | 0.664 | 1.000 | 0.115 | 0.705 | 0.157 | 0.514 | 0.156 | 0.587 |
| NPE11 | 0.251 | 0.507 | 0.724 | 0.703 | 0.038 | 0.582 | 0.977 | 0.349 | 0.335 | 0.755 | 0.434 | 0.455 | 0.488 | 0.695 |
| NPE12 | 0.216 | 0.458 | 0.708 | 0.829 | 0.177 | 0.604 | 0.782 | 0.292 | 0.162 | 0.651 | 0.326 | 0.499 | 0.413 | 0.648 |
| PE1 | 1.000 | 1.000 | 0.578 | 1.000 | 0.642 | 0.239 | 0.901 | 0.048 | 0.922 | 0.792 | 0.908 | 0.788 | 0.826 | 0.927 |
| PE2 | 0.781 | 0.934 | 0.800 | 0.847 | 0.943 | 0.418 | 0.839 | 0.108 | 0.380 | 0.732 | 0.725 | 0.885 | 0.690 | 0.865 |
| PE3 | 0.599 | 0.846 | 0.730 | 0.694 | 0.716 | 0.510 | 0.790 | 0.162 | 0.236 | 0.792 | 0.698 | 0.821 | 0.753 | 0.900 |
| PE4 | 0.549 | 0.813 | 0.659 | 0.757 | 0.601 | 0.329 | 0.893 | 0.128 | 0.460 | 0.632 | 0.545 | 0.770 | 0.516 | 0.788 |
| PE5 | 0.332 | 0.659 | 0.735 | 0.811 | 0.482 | 0.307 | 0.870 | 0.062 | 0.475 | 0.793 | 1.000 | 0.756 | 1.000 | 1.000 |
| PE6 | 0.389 | 0.758 | 0.838 | 0.946 | 0.826 | 0.102 | 0.977 | 0.019 | 1.000 | 0.482 | 0.666 | 0.995 | 0.645 | 0.802 |
| PE7 | 0.483 | 0.813 | 0.789 | 0.838 | 0.421 | 0.332 | 0.979 | 0.090 | 0.519 | 0.626 | 0.563 | 0.688 | 0.545 | 0.712 |
| PE8 | 0.226 | 0.423 | 0.686 | 0.622 | 0.030 | 0.451 | 0.993 | 0.115 | 0.505 | 1.000 | 0.400 | 0.611 | 0.018 | 0.780 |
| PE9 | 0.555 | 0.733 | 0.659 | 0.838 | 1.000 | 0.151 | 0.900 | 0.031 | 0.667 | 0.585 | 0.802 | 1.000 | 0.793 | 0.891 |

**Supplementary Information s5: Further Discussion on Classification Analysis presented**

**Supervised Learning:**

The classification will use the two-fold cross-validation. The data is split in training data and testing data as 90% will be used for training and 10% for testing. As the dataset contains only 21 patients, a higher percentage of the dataset was allocated to training. The testing set includes 2 patients from each group and their values for each classifier was close to the mean of the group. However, only 2 patients will affect the testing and the assessment of the predictive ability of the model. This process has been repeated ~5 times with a combination of patients selected for testing to ensure the reliability of the model and understand the variation. Due to the reduced variation between iterations. The reported results (Table 1) include the accuracy/sensitivity/specificity of both Training and Testing stages while the AUC is based solely on the training data.

The decision to present the results only on one supervised learning model has been taken after a full analysis was completed on 28 supervised machine learning (ML) algorithms (all algorithms can be found in the Classification Learner App in MATLAB R2021b but a few examples are: Linear Discriminant (LD), Decision Trees, Support Vector Machine (SVM), Naive Bayes, k-nearest Neighbour (KNN) and Neural Networks).

The analysis also included the combinations of multiple biomarkers to understand if the classification improves (up to 3 biomarkers). The majority of the algorithms exhibited comparable outcomes, and the integration of various biomarkers did not enhance the classification performance relative to the use of individual biomarkers.

Logistic Regression algorithm has presented one of the best results in classification of the two groups, with results similar to LD, Decision Trees, and SVM (the average standard deviation for accuracy of all algorithms was less than 10%).

**Unsupervised Learning:**

Unsupervised ML is using k-means as the sole technique for classification. K-means clustering is one the most popular and simple techniques used in unsupervised ML. It groups the data points in k clusters and tries to identify any underlying patterns. The cluster is formed based on the distance from the centroid. The centroid is found by using iterative calculations: first, the centroids are allocated randomly and from iterative calculations, the centroids locations get optimised until a stable location is found. In this work, k was set to 2, the distance metric used is ‘cityblock’ (each element is the component wise median of the points in that cluster), the maximum number of iterations was set to 1000 and the number of times to repeat clustering using new initial centroid position was set to 20. Other variations of these settings were tried but the difference between clustering was insignificant. The A, CI, SE, and SP were calculated for k-means.

**Supplementary Information s6: Further Discussion on Computational model**

The model consists of 513 1D vessels and 62 vascular beds encompassing all bodily circulations such as the pulmonary circulation, the cerebral circulation, the coronary circulation, the hepatic-portal circulation and especially the utero-ovarian circulation. The vascular beds cover organs like the brain, stomach, spleen, liver, intestines, right and left kidneys, and body parts such as left and right shoulder, arms, legs, chest, face and others. Vascular beds significant in pregnancy include the uterus, placenta, ovaries, and cervix. The flow through each compliant major vessel is described by a set of partial differential equations that conserve mass and momentum. Hence, the model satisfies the first principles of physics to calculate the pressures and flows throughout the vascular system.

The model adapts to patient data using an quasi-Newton optimisation approach, with heart rate and patient height are input parameters of the model. The model then iteratively compares model outputs (systolic/diastolic pressure, cardiac output) against the measured data and adjusts the peripheral resistances, arterial compliances and blood volume until convergence is reached. Subsequently, the model is simplified to the arterial system and transformed in an open-loop forward model. This second loop uses the initial conditions from the first loop to adjust parameters until systolic/diastolic pressure and arterial pulse wave velocity match patient data. Finally, it adapts the uterine, arcuate, and radial arteries vessel diameter to match measured Doppler scan velocities. The reader is referred to [24], [26] and [27] for a detailed technical description of the computational framework.

**Supplementary Information s7: Comparison of Proposed Biomarker Performance**

To assess the different biomarker performances we have used DeLong’s test, which compares the AUC between models. The below matrix shows the p-values between the different biomarkers evaluated in this manuscript.


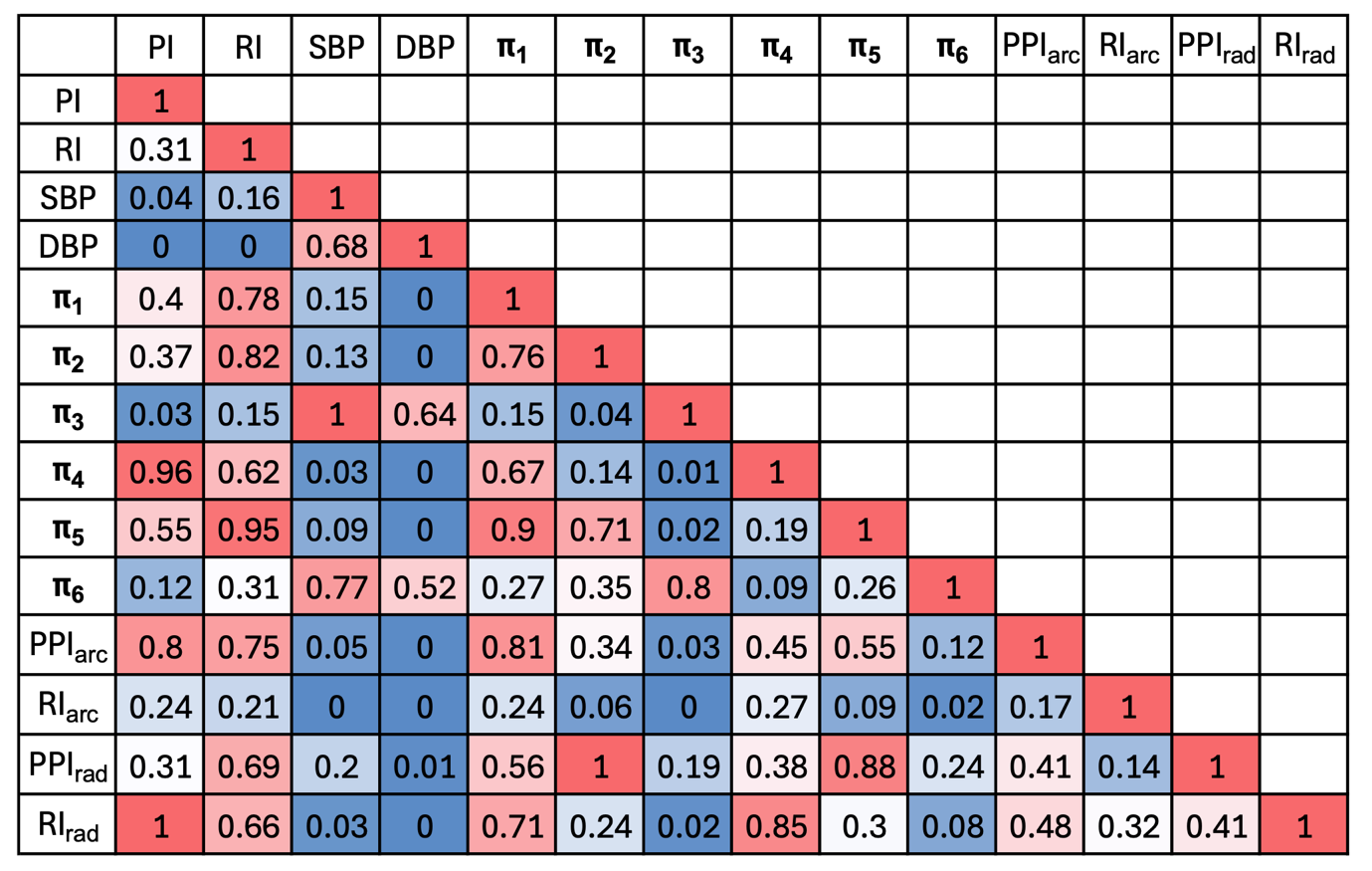

Supplement: Supplementary file 1 — Supplementary Material 1. [file 41598_2024_72832_MOESM1_ESM.doc]
